# Recombinant Lactococcus Expressing a Novel Variant of Infectious Bursal Disease Virus VP2 Protein Can Induce Unique Specific Neutralizing Antibodies in Chickens and Provide Complete Protection

**DOI:** 10.3390/v12121350

**Published:** 2020-11-25

**Authors:** Zhihao Wang, Jielan Mi, Yulong Wang, Tingting Wang, Xiaole Qi, Kai Li, Qing Pan, Yulong Gao, Li Gao, Changjun Liu, Yanping Zhang, Xiaomei Wang, Hongyu Cui

**Affiliations:** State Key Laboratory of Veterinary Biotechnology, Harbin Veterinary Research Institute, Chinese Academy of Agricultural Sciences, Harbin 150069, China; a17854265231@163.com (Z.W.); mijielan1007@163.com (J.M.); xyylong@126.com (Y.W.); wangtingting0852@163.com (T.W.); qxl@hvri.ac.cn (X.Q.); likaihvri@163.com (K.L.); panqing@caas.cn (Q.P.); ylgao8@163.com (Y.G.); gaoli0820@163.com (L.G.); liucj93711@hvri.ac.cn (C.L.); zhangyanping03@caas.cn (Y.Z.)

**Keywords:** infectious bursal disease virus, immunization, recombinant Lactococcus lactis, variant strain, vaccine

## Abstract

Recent reports of infectious bursal disease virus (IBDV) infections in China, Japan, and North America have indicated the presence of variant, and the current conventional IBDV vaccine cannot completely protect against variant IBDV. In this study, we constructed recombinant *Lactococcus lactis* (r-*L. lactis*) expressing a novel variant of IBDV VP2 (avVP2) protein along with the Salmonella resistance to complement killing (RCK) protein, and Western blotting analysis confirmed that r-*L. lactis* successfully expressed avVP2-RCK fusion protein. We immunized chickens with this vaccine and subsequently challenged them with the very virulent IBDV (vvIBDV) and a novel variant wild IBDV (avIBDV) to evaluate the immune effect of the vaccine. The results show that the r-*L. lactis*-avVP2-RCK-immunized group exhibited a 100% protection rate when challenged with avIBDV and 100% survival rate to vvIBDV. Furthermore, this immunization resulted in the production of unique neutralizing antibodies that cannot be detected by conventional ELISA. These results indicate that r-*L. lactis*-avVP2-RCK is a promising candidate vaccine against IBDV infections, which can produce unique neutralizing antibodies that cannot be produced by other vaccines and protect against IBDV infection, especially against the variant strain.

## 1. Introduction

Infectious bursal disease virus (IBDV), also known as Gumboro, is the causative agent of a highly infectious disease in chickens. The main features of infectious bursal disease (IBD) is an infection of the central organ of the immune system and the damage of B lymphocytes in the bursa of Fabricius [1,2]. IBD can cause strong immunosuppression in chickens [3], affect the immunological effects of multiple vaccines, such as Newcastle disease, Avian infectious bronchitis, and Chicken Infectious Anemia, and increase the infection rates of bacteria such as *Escherichia coli*, *Salmonella*, and *Staphylococcus aureus* [4,5,6]. Hence, IBD has received significant attention from the poultry industry, where chickens are protected by vaccination [1]. However, recent reports have suggested that most of the IBDVs popular in Japan, North America, and China were variant IBDVs, and caused huge economic losses [7,8,9,10,11]. Since conventional commercial vaccines cannot provide complete protection against such variant strains [9,12], there is an urgent need to develop a variant IBDV vaccine.

Although attenuated live vaccines offer good prospects for the prevention and treatment of very virulent IBDV (vvIBDV), live IBD vaccines present risks of virulence enhancement and immunosuppression if they are widely used [3,13]. Therefore, these issues have generated increasing interest in the development of subunit vaccines. The neutralizing escape epitope located in the highly variable region of the VP2 protein in IBDV can induce the body to produce neutralizing antibodies to protect the host from IBDV infection [14,15]. VP2 was found to be expressed as a target antigen protein in baculoviruses [16,17], *E. coli* [18,19], yeasts [20,21], and plant and insect cell lines [22]. Thus, VP2 is commonly purified or processed to form a virus-like particle (VP2-VLP) [23], which provides complete immune protection to chickens against IBDV upon immunization [16,24,25,26]. Our previous studies showed that a recombinant Lactococcus co-expressing the outer membrane protein (Omp) H of the microfold (M) cell-targeting ligand and the major vvIBDV antigens VP2 and a recombinant Lactococcus co-expressing the major vvIBDV antigens VP2 and resistance to complement killing (RCK) protein of Salmonella enterica were promising candidate vaccines to prevent vvIBDV infection [24,25]. However, live vaccines and inactivated vaccines against vvIBDV, vvIBDV VP2 subunit vaccine cannot completely protect against the variant IBDV strains [12]. Therefore, in this study, we designed a subunit vaccine against the variant IBDV strain.

We used *Lactococcus lactis* (*L. lactis*) as the host strain for producing recombinant proteins. Lactic acid bacteria are Gram-positive bacteria that can ferment carbohydrates to lactic acid [27]. *L. lactis* is a food-grade probiotic with non-pathogenic, non-invasive, and non-colonizing properties. Therefore, *L. lactis* is an ideal host for the production of recombinant proteins [28,29]. As a promising candidate for use as antigen carriers [30,31], a major advantage of this system is the ability to safely deliver antigens to the immune system [32]. Therefore, we used *L. lactis* as a vector to express heterologous proteins, which is a common practice [24,25,33,34,35,36,37,38]. Moreover, to enhance the antigen presentation of avVP2 and improve the immune protection efficiency, we utilized fusion expression avVP2-RCK following our previous study. In our previous study, we co-expressed vvIBDV-VP2-RCK fusion protein and detected high levels of specific neutralizing antibodies against vvIBDV after immunization [24].

In this study, we report the expression of a novel variant IBDV (avIBDV) antigen avVP2-RCK fusion protein in *L. lactis*. Using the nisin-controlled gene expression system, recombinant *L. lactis* was used for injection immunization of chickens. We found that r-*L. lactis*-avVP2-RCK could induce the body to produce a high level of unique specific neutralizing antibodies, which provided complete immune protection against avIBDV, and the survival rate could reach 100% after the challenge with vvIBDV.

## 2. Materials and Methods

### 2.1. Experimental Materials

*L. lactis* NZ3900 and the expression vector pNZ8149 were procured from MoBiTec (MoBiTec, Goettingen, Germany). Chinese vvIBDV reference strain HLJ0504 (GenBank accession: GQ451330 (Segment A); GQ451331 (Segment B), vvIBDV-HLJ0504) and a novel variant IBDV wild strain SHG19 (GenBank accession: MN393076 (Segment A); MN393077 (Segment B), avIBDV-SHG19) were stored at the Harbin Veterinary Research Institute (HVRI) of the Chinese Academy of Agricultural Sciences (CAAS) at −70 °C [39]. VP2 specific mouse monoclonal antibody (MAb) was prepared in our laboratory according to standard procedures [40]. Commercial live vaccine Gt (the licensed attenuated live vaccine) was purchased from the Weike Biotechnology Development Company of China, and commercial live vaccine B87 (the licensed medium virulent live vaccine) was purchased from the Howe Biotechnology Company of China. Lipopolysaccharide (LPS) was purchased from Sigma (Sigma-Aldrich, St. Louis, MO, USA). Phorbol 12-myristate 13-acetate (PMA) and concanavalin A (ConA) were purchased from Invivogen (Invivogen, Toulouse, France). Cell Counting Kit-8 was purchased from Dojindo (Dojindo Laboratories, Kumamoto, Japan).

### 2.2. Construction of Recombinant Plasmid and Cell Transformation

The avVP2-RCK gene was amplified using the forward primer 5′-AGGCACTCACCATGACAAATTTAC-3′ and the reverse primer 5′-GTTCAAAGAAAGCTTAAACAACATT-3′ from the plasmid pUC57-avIBDV-VP2-RCK (kindly codon-optimized and synthesized by the Nanjing GenScript Biotechnology Corporation, China). The linear fragment of pNZ8149 was amplified by PCR using the forward primer 5′- GCTTTCTTTGAACCAAAATTAG-3′ and the reverse primer 5′-GGTGAGTGCCTCCTTATAATTTATT-3′. Homologous recombination of the avVP2 fragment and pNZ8149 linear vector was performed using a one-step cloning kit (Vazyme Biotech Co., Nanjing, China). The recombinant vector pNZ8149-avVP2-RCK was transformed into *L. lactis* NZ3900 by electroporation and r-*L. lactis*-avVP2-RCK was selected on Elliker culture agar plates supplemented with 0.5% lactose, and grown at 30 °C according to standard protocols [41]. Subsequently, avVP2-RCK fragments were detected by PCR to identify positive strains.

### 2.3. Nisin-Induced Expression and Western Blotting Analysis

Nisin-induced expression system was used according to the protocol described previously [24,25]. Briefly, the recombinant lactic acid bacteria cultured overnight were inoculated in Elliker culture solution at a 1:100 ratio and cultured at 30 °C until the optical density at 600 nm (OD600) reached values of 0.4 to 0.5. Nisin (10 ng/mL) was then added to induce expression for 4 to 5 h. After centrifugation at 8000× *g* for 5 min, the cells were resuspended at a 1:10 ratio in sterile water, and the cells were destroyed with the EpiShear probe sonicator for subsequent analysis. The same method was followed for the r-*L. lactis*-pNZ8149 control.

Western blotting was performed to verify the expression of the target protein as described previously [24,25]. In short, the ultrasonically broken protein samples were electrophoretically separated by 8–12% SDS-PAGE. Subsequently, mouse monoclonal anti-VP2 antibody and IRDye 800CW goat anti-mouse secondary antibody (LI-COR Biosciences, Lincoln, NE, USA) were used for detection. Finally, we used an Odyssey imaging system (LI-COR Biosciences) to analyze the protein bands.

### 2.4. Recombinant VP2 Protein Quantitative Analysis

To quantify the content of avVP2-RCK in the supernatant, a double dilution standard of 2 mg/mL bovine serum albumin was used. The r-*L. lactis*-pNZ8149 lysate (negative control) and r-*L. lactis*-avVP2-RCK lysate (sample) were electrophoretically separated by 8–12% SDS-PAGE, and the gel was stained with Coomassie Brilliant Blue. By drawing the relationship between the gray value of the gradient bovine serum albumin on the *x*-axis and the concentration value on the *y*-axis, an optimal standard curve was generated. The recombinant protein concentration was calculated after subtracting the background effect of the negative control [25].

### 2.5. Experimental Chickens

Specific-pathogen-free (SPF) chickens were purchased from the Experimental Animal Center of the Harbin Veterinary Research Institute of the Chinese Academy of Agricultural Sciences (HVRI of CAAS, Harbin, China) and raised in a negative-pressure isolator fitted with an air filter. All animal experiments were approved by the HVRI of CAAS and carried out by animal ethics guidelines and approved protocols (SYXK (Hei) 2017-009). In this study, the animal experimental permission date was 04-23-2020, and the number of experimental animals was 93.

### 2.6. Immunoprotection Experiment

r-*L. lactis*-avVP2-RCK was inoculated and induced with nisin as described above. The collected cells were washed twice with sterile phosphate buffer saline (PBS) and then suspended in sterile PBS to immunize at an appropriate concentration (1 × 10^9^ CFU/mL r-*L. lactis*-avVP2-RCK and controls in a 500 μL volume) and then inactivated at 70 °C for 10 min.

The 15-day-old SPF chickens were randomly divided into four groups. In group one, 23 chickens were immunized with 500 μL inactivated r-*L. lactis*-avVP2-RCK by intramuscular injection. In group two, 20 chickens were immunized with the Gt live vaccine by intraocular-nasal route. In group three, 20 chickens were immunized with the B87 live vaccine by intraocular-nasal route. In group four, 30 chickens were used as a non-immunized healthy control group. After 15 days of immunization, SPF chickens in each group were regrouped to challenge with Chinese vvIBDV reference strain HLJ0504 (vvIBDV-HLJ0504) and a novel variant IBDV wild strain SHG19 (avIBDV-SHG19). In group one, 10 chickens were challenged with vvIBDV-HLJ0504, 10 chickens challenged with avIBDV-SHG19, and 3 were not challenged with any virus. In groups two and three, 10 chickens from each group were challenged with vvIBDV-HLJ0504, and 10 chickens from each group were challenged with avIBDV-SHG19. In group four, 10 chickens were challenged with vvIBDV-HLJ0504, 10 chickens were challenged with avIBDV-SHG19, and 10 chickens were not challenged with the virus. The challenge dose of vvIBDV-HLJ0504 was 10^3^ ELD_50_ (median embryo lethal dose, ELD_50_) and the challenge dose of the avIBDV-SHG19 was 10 BAD_50_ (50% buysae atrophy dose, BAD_50_). After 7 days of challenge, the surviving animals were put to death, and autopsies were performed. We then isolated peripheral blood mononuclear cells (PBMCs) from fresh anticoagulant blood to detect their proliferative activity. The bursa/body weight index (BBIX) and bursa/body weight were calculated as described previously [25]. Serums were collected two weeks and three weeks after immunization, and one week after challenge, and then stored at −80 °C.

### 2.7. Serological ELISA Antibody Detection and Neutralization Test

Neutralization tests to detect serum neutralizing antibodies have been described previously [9,42]. Briefly, the serum collected after two weeks of immunization was diluted twice (briefly, 100 μL of serum was mixed with 100 μL of RPMI 1640 culture medium in the first hole, then 100 μL of the mixture was mixed with 100 μL of RPMI 1640 culture medium in the second hole, starting from 1:2^1^ to 1:2^12^), and 100 μL of the diluted serum was mixed with 100 μL of 200 TCID_50_ (50% tissue culture infective dose, TCID_50_) vvIBDV-HLJ0504 or avIBDV-SHG19 and incubated for 1 h at 37 °C in a cell culture incubator. Thereafter, this serum and virus mixture (100 μL) was added to DT40 cells and incubated at 37 °C for 24 h. The infection-positive wells were detected by immunofluorescence assays. Meanwhile, the serum samples after two weeks of immunization were tested with IBDV VP2-coated ELISA kit (IDEXX IBD-XR Ab Tests kit, Westbrook, ME, USA) and IBD whole virus-coated ELISA (IDEXX IBD Ab Tests kit, Westbrook, ME, USA).

### 2.8. Measurement of PBMC Cell Proliferation Activity

The proliferative activity of PBMCs stimulated by concanavalin A (ConA) and Phorbol 12-myristate 13-acetate (PMA) was measured as described previously [42]. Isolated chicken PBMCs were obtained using a peripheral chicken blood mononuclear cell isolate kit (TBD, Tianjin, China). PBMCs were diluted to 1 × 10^6^ cells/mL with RPMI 1640 culture medium containing 10% fetal bovine serum (Gibco, Gaithersburg, MD, USA) and penicillin-streptomycin (Wisent, Saint-Jean-Baptiste, QC, Canada). Then, 100 μL PBMCs were added to each well of the 96-well plates. Next, 5 μg/mL ConA and 100 ng/mL PMA were added to each well and maintained at 37 °C in a cell incubator for 48 h. Subsequently, 10 μL CCK-8 was added for 4 h, and absorbance was measured at 450 nm.

The proliferative activity of PBMCs stimulated with Lipopolysaccharide (LPS) was measured as described previously [43]. PBMC was diluted to 1 × 10^6^ cells/mL with RPMI 1640 culture medium containing 10% fetal bovine serum (Gibco, Gaithersburg, MD, USA) and penicillin-streptomycin (Wisent, Saint-Jean-Baptiste, QC, Canada). Subsequently, 100 μL of this solution were added to a 96-well plate, along with LPS at a final concentration of 1 μg/mL per well, and maintained at 37 °C for 72 h in a cell culture incubator. Next, 10 μL CCK-8 were added for 4 h, and absorbance was measured at 450 nm.

### 2.9. Cytokine ELISA

The serum collected two weeks after immunization and one week after challenge with the virus was diluted to a 1:2 ratio, and interleukins (IL) IL-4, IL-10, and IL-12, and interferon-γ (IFN-γ) were detected using Indirect fluorescence ELISA kit (Solarbio, Beijing, China) according to the manufacturer’s instructions. Cytokine concentration was calculated according to the standard curve.

### 2.10. Statistical Analysis

Data were analyzed using Prism 6 software (GraphPad Inc., La Jolla, CA, USA). One-way ANOVA was used to evaluate the statistical significance of the differences among different groups, and results with *p* < 0.05 were considered statistically significant.

## 3. Result

### 3.1. Construction of Recombinant Lactic Acid Bacteria and Expression and Quantitative Analysis of Target Protein

**Figure 1:**
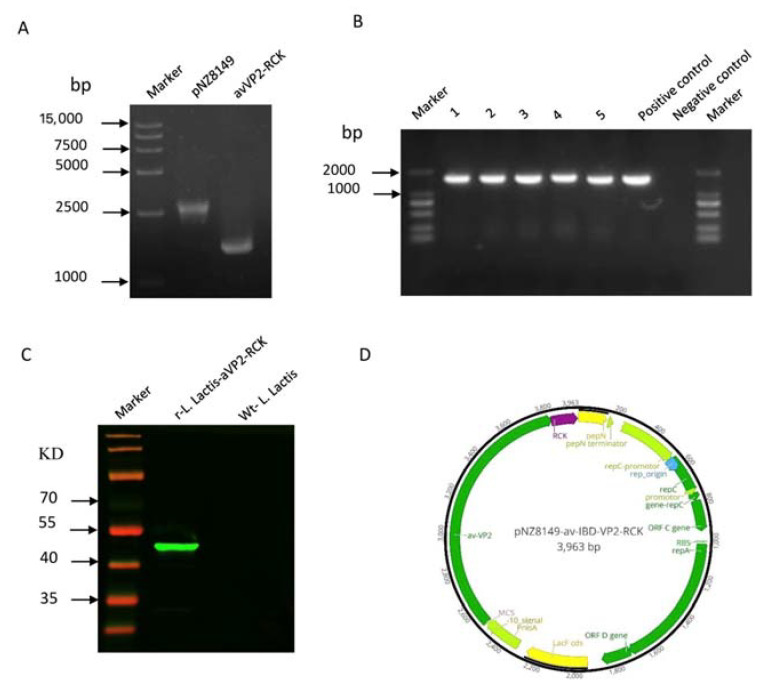
Construction of the plasmid pNZ8149-avVP2-RCK expressing the avVP2-RCK fusion protein and identification of recombinant proteins via Western blotting analysis. (**A**) PCR amplification of pNZ8149 (2505 bp, lane 2) and avVP2-RCK (1491 bp, lane 3) optimization gene. (**B**) Amplified avVP2-RCK fragments (1491 bp, lanes 2–6) by bacterial solution PCR to screen positive strains. (**C**) Immunoblot analysis of total whole-cell protein extracts from r-*L. lactis*-avVP2-RCK. (**D**) Schematic diagrams of the recombinant plasmid pNZ8149-avVP2-RCK with the avVP2-RCK gene fusion.

First, we amplified the 2505 bp pNZ8149 linear vector and the 1491 bp avVP2-RCK target fragment with homology arms (Figure 1A Lanes 2 and 3), and then transferred the recombinant plasmid into *L. lactis*. The r-*L. lactis* expressing the target fragment avVP2-RCK were successfully screened by PCR (Figure 1B Lanes 2–6). After sonicating the r-*L. lactis*, the expression of target protein avVP2-RCK (about 52 kDa) was verified in r-*L. lactis*-avVP2-RCK NZ3900 (Figure 1C, Lane 2; Figure 1D), whereas the target protein avVP2-RCK was not expressed in the empty vector strain (Figure 1C Lane 3). Quantitative analysis showed that the expression level of the target protein was 55.4 μg/mL in r-*L. lactis*-avVP2-RCK.

### 3.2. Recombinant Lactococcus Induces Unique and Specific Neutralizing Antibodies

**Figure 2:**
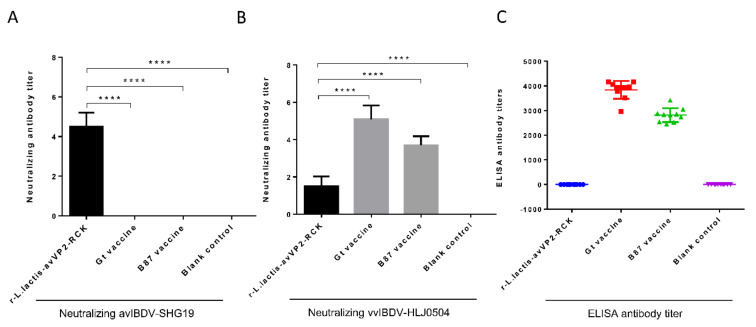
Serum antibody response after two weeks of immunization. Detection and comparison of virus neutralization antibodies against avIBDV-SHG19 (**A**) and vvIBDV-HLJ0504 (**B**) in chickens. (**C**) Detection of Elisa antibodies against IBDV virus in chickens two weeks after vaccination. Statistical significance was set at **** *p* < 0.0001. IBDV: infectious bursal disease virus.

Two weeks after immunization, the level of the neutralizing antibody of the r-*L. lactis*-avVP2-RCK group serum against avIBDV-SHG19 reached 1:2^4−6^ (Figure 2A), extremely significantly higher than the Gt group (1:2^0^), B87 Group (1:2^0^), and the blank control group (1:2^0^) (*p* < 0.0001) (Figure 2A). The level of neutralizing antibodies of the r-*L. lactis*-avVP2-RCK group serum against vvIBDV-HLJ0504 reached 1:2^1−2^ (Figure 2B), which was extremely significantly lower than the Gt group (1:2^4−6^) (*p* < 0.0001) and B87 group (1:2^3−4^) (*p* < 0.0001), but extremely significantly higher than the blank control group (1:2^0^) (*p* < 0.0001) (Figure 2B).

Of particular importance is that the serum antibody titers in the r-*L. lactis*-avVP2-RCK group is negative when using the whole virus coated ELISA (Figure 2C), while the serum antibody titers in the Gt group reached close to 4000 (Figure 2C). The ELISA serum antibody titers in the B87 group reached approximately 3000 (Figure 2C); similar results were obtained using the IBDV VP2-coated ELISA (data not shown), and we repeated the ELISA test multiple times and got the same results. The above results show that injection of r-*L. lactis*-avVP2-RCK can produce a specific immune response against the avIBDV-VP2, but it induces a unique and specific neutralizing antibody.

### 3.3. Immunoprotective Effects against vvIBDV and avIBDV

**Figure 3:**
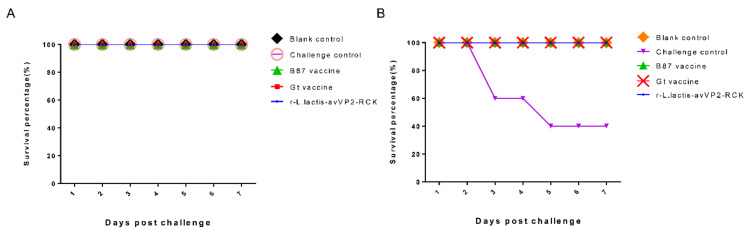
Protective efficacy of every group against avIBDV-SHG19 (**A**) and vvIBDV-HLJ0504 (**B**) in immunized chicken and survival rate over 7 days.

**Figure 4:**
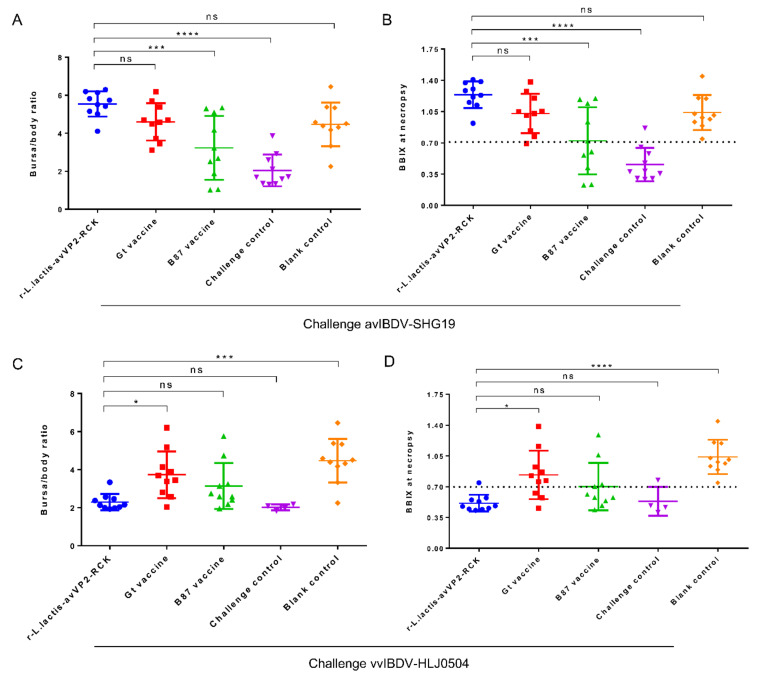
Analysis of protection against IBDV challenge determined by calculating BBIX values and bursa/body ratio. Bursa/body ratio (**A**) and BBIX (**B**) after challenge avIBDV-SHG19. (**C**,**D**) Bursa/body ratio (**C**) and BBIX (**D**) after challenge vvIBDV-HLJ0504. Surviving birds were euthanized and analyzed after the observation period on day 7 post challenge. Statistical significance was set at * *p* < 0.05, *** *p* < 0.001, **** *p* < 0.0001. NS, no significance; BBIX, bursa/body weight index.

One week after being challenged with avIBDV-SHG19, the survival rates of chickens from the r-*L. lactis*-avVP2-RCK, Gt live vaccine, B87 live vaccine, blank control, and challenge control groups were all 100% (Figure 3A). Necropsy revealed that the bursa/body ratio of chickens from the r-*L. lactis*-avVP2-RCK group was extremely significantly higher than that of chickens from the B87 live vaccine group (*p* < 0.001) and the challenge control group (*p* < 0.0001). However, no significant difference was observed in the bursa/body ratio of chickens from the r-*L. lactis*-avVP2-RCK group, and those from the Gt live vaccine group and the blank control group (*p* > 0.05) (Figure 4A). The BBIX of all chickens in the r-*L. lactis*-avVP2-RCK group was greater than 0.7 (Figure 4B), which was extremely significantly higher than that of chickens from the B87 live vaccine group (*p* < 0.001) and the challenge control group (*p* < 0.0001). However, the index of the r-*L. lactis*-avVP2-RCK group chickens was not significantly different from that of the Gt live vaccine group and the blank control group chickens (*p* > 0.05) (Figure 4B). These results show that r-*L. lactis*-avVP2-RCK immunization can completely protect against avIBDV-SHG19.

One week after the challenge of vvIBDV-HLJ0504, the survival rates of chickens from the r-*L. lactis*-avVP2-RCK, Gt live vaccine, B87 live vaccine, and blank control groups were 100%, while the survival rate of the chickens from the challenge control group was 40% (Figure 3B). Necropsy revealed that the bursa/body ratio of chickens from the r-*L. lactis*-avVP2-RCK group was extremely significantly lower than that of chickens from the blank control group (*p* < 0.001) and significantly lower than that of the Gt live vaccine group (*p* < 0.05). However, the bursa/body ratio of chickens of the r-*L. lactis*-avVP2-RCK group was not significantly different from that of chickens from the B87 blank control group or the challenge control group (*p* > 0.05) (Figure 4C). The BBIX of chickens from the r-*L. lactis*-avVP2-RCK group was less than 0.7 (Figure 4D), which was extremely significantly lower than that of chickens from the blank control group (*p* < 0.0001); significantly lower than the Gt live vaccine group (*p* < 0.05). However, it was not significantly different from chickens from the B87 blank control group and the challenge control group (*p* > 0.05) (Figure 4D). These results suggest that, although the survival rate of the immunized group was 100%, immunization did not protect the bursa of these chickens.

### 3.4. Peripheral PBMCs Proliferation Activity

**Figure 5:**
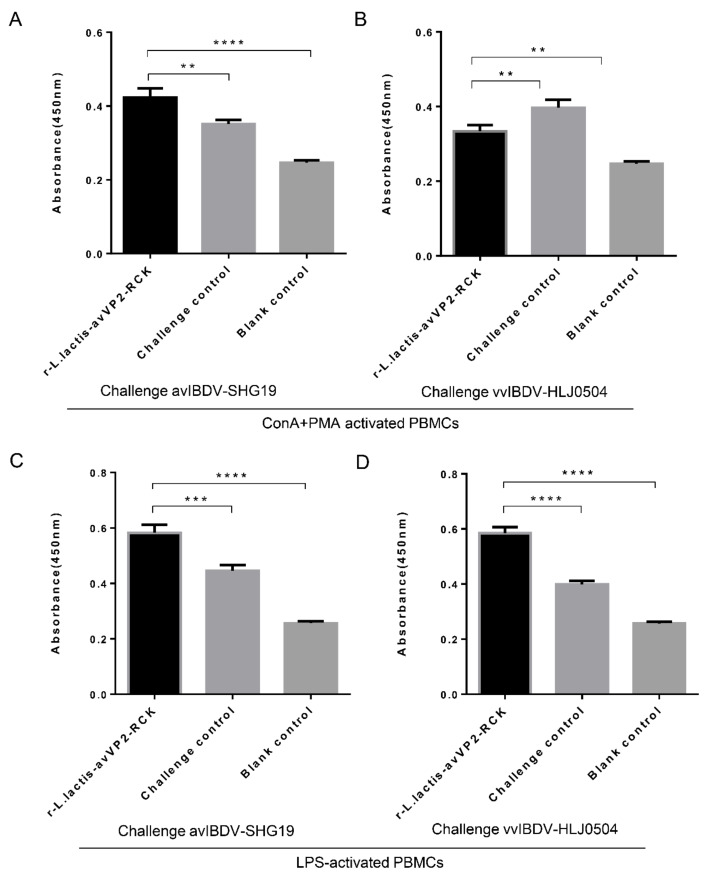
The peripheral PBMCs in ConA+PMA; LPS-stimulated cell proliferation activity after two weeks after immunization and one week of challenge IBDV. The peripheral PBMCs in ConA+PMA-stimulated cell proliferation activity after challenge avIBDV-SHG19 (**A**) and vvIBDV-HLJ0504 (**B**). The peripheral PBMCs in LPS-stimulated cell proliferation activity after challenge avIBDV-SHG19 (**C**) and vvIBDV-HLJ0504 (**D**). Statistical significance was set at ** *p* < 0.01, *** *p* < 0.001, **** *p* < 0.0001.

PBMCs collected two weeks after immunization and one week after challenge were stimulated by ConA and PMA. The results showed that the cell proliferation activity of PBMCs was not significantly different among the different groups after two weeks of immunization (*p* > 0.05). After challenge with avIBDV-SHG19 for one week, cell proliferation activity of peripheral PBMCs of the r-*L. lactis*-avVP2-RCK group was significantly higher than that of the challenge control group (*p* < 0.01) and the blank control group (*p* < 0.0001) (Figure 5A). After challenge with vvIBDV-HLJ0504, cell proliferation activity of peripheral PBMCs in the r-*L. lactis*-avVP2-RCK group was significantly lower than that of the challenge control group (*p* < 0.01) and significantly higher than that of the blank control group (*p* < 0.01) (Figure 5B). These results show that r-*L. lactis*-avVP2-RCK can significantly increase the proliferation activity of specific T cells in PBMCs, which is beneficial for the immune response against infection.

PBMCs collected two weeks after immunization and one week after challenge were also stimulated by LPS. The results showed that the cell proliferation activity of PBMCs was not significantly different among the different groups after two weeks of immunization (*p* > 0.05). After challenge with avIBDV-SHG19 for one week, the cell proliferation activity of peripheral PBMCs in the r-*L. lactis*-avVP2-RCK group was extremely significantly higher than that of the challenge control group (*p* < 0.001) and the blank control group (*p* < 0.0001) (Figure 5C). After challenge with vvIBDV-HLJ0504, the proliferation activity of peripheral PBMCs in the r-*L. lactis*-avVP2-RCK group was extremely significantly higher than that of the challenge control group (*p* < 0.0001) and the blank control group (*p* < 0.0001) (Figure 5D). Thus, these results show that immunization with r-*L. lactis*-avVP2-RCK can significantly increase the proliferation activity of antigen-presenting cells in PBMCs, which is beneficial for the immune response against infection.

### 3.5. ELISA Detects Serum Cytokines

**Figure 6:**
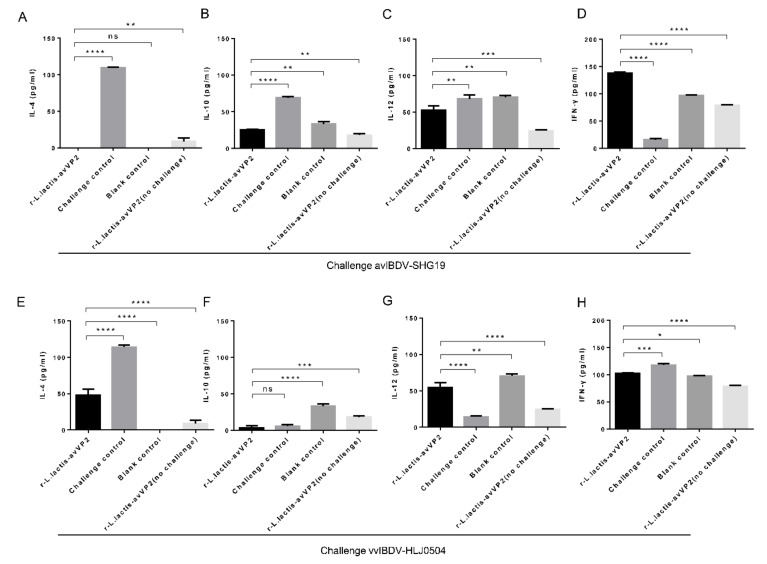
Elisa was used to detect cytokines in serum collected one week after challenge. Detection of IL-4 (**A**), IL-10 (**B**), IL-12 (**C**), and IFN-γ (**D**) antibody in serum after challenge variant IBDV-SHG19. Detection of IL-4 (**E**), IL-10 (**F**), IL-12 (**G**), and IFN-γ (**H**) after challenge vvIBDV-HLJ0504. Statistical significance was set at * *p* < 0.05, ** *p* < 0.01, *** *p* < 0.001, **** *p* < 0.0001. NS, no significance.

Serum cytokines were tested one week after the challenge with avIBDV-SHG19, and the results showed that the level of IL-4 in the serum of the r-*L. lactis*-avVP2-RCK group was extremely significantly lower than that of the challenge control group (*p* < 0.0001), the immune non-challenge control group (*p* < 0.01), but not significantly different from that of the blank control group (*p* > 0.05) (Figure 6A). However, the level of IL-10 in the serum of the r-*L. lactis*-avVP2-RCK group was extremely significantly lower than that of the challenge control group (*p* < 0.0001) and significantly lower than the blank control group (*p* < 0.01), but significantly higher than that of the immune non-challenge control group (*p* < 0.01) (Figure 6B). The level of IL-12 in the serum of the r-*L. lactis*-avVP2-RCK group was significantly lower than that of the challenge control group (*p* < 0.01) and the blank control group (*p* < 0.01), but extremely significantly higher than that of the immune non-challenge control group (*p* < 0.001) (Figure 6C). Similarly, the level of IFN-γ in the serum of r-*L. lactis*-avVP2-RCK group was extremely significantly higher than that of the challenge control group (*p* < 0.0001), the blank control group (*p* < 0.0001), and the immune non-challenge control group (*p* < 0.0001) (Figure 6D). These results show that vaccination with r-*L. lactis*-avVP2-RCK significantly improved the immune response against infection without causing adverse inflammatory reactions.

The serum cytokines were also tested one week after challenge with vvIBDV-HLJ0504. The results showed that the level of IL-4 in the serum of the r-*L. lactis*-avVP2-RCK group was extremely significantly lower than that of the challenge control group (*p* < 0.0001), but extremely significantly higher than that of the blank control group (*p* < 0.0001) and the immune non-challenge control group (*p* < 0.0001) (Figure 6E). When we examined IL-10, we found that the level of IL-10 in the serum of the r-*L. lactis*-avVP2-RCK group was extremely significantly lower than that of the blank control group (*p* < 0.0001) and the immune non-challenge control group (*p* < 0.001), but not significantly different from that of the challenge control group (*p* > 0.05) (Figure 6F). The level of IL-12 in the serum of the r-*L. lactis*-avVP2-RCK group was extremely significantly higher than that of the challenge control group (*p* < 0.0001) and the immune non-challenge control group (*p* < 0.0001), but significantly lower than that of the blank control group (*p* < 0.01) (Figure 6G). In contrast, the level of IFN-γ in the serum of the r-*L. lactis*-avVP2-RCK group was extremely significantly lower than that of the challenge control group (*p* < 0.001), but significantly higher than that of the blank control group (*p* < 0.05), and extremely significantly higher than the immune non-challenge control group (*p* < 0.0001) (Figure 6H). These results show that the immunization with r-*L. Lactis*-avVP2-RCK can also improve the anti-infective immune response to typical virulent toxins without causing adverse inflammation.

## 4. Discussion

In this research, a very important discovery was that the injection of inactivated r-*L. lactis*-avVP2-RCK into SPF chickens induces to produce unique specific neutralizing antibodies, which provide complete protection of avIBDV-SHG19. However, more importantly, this unique neutralizing antibody cannot be detected by conventional IBD antibody ELISA kits, and ELISA cannot detect unique neutralizing antibodies. In contrast, many live IBDV vaccines, inactivated vaccines, and VP2-VLP subunit vaccines can produce detectable IBDV ELISA antibodies in the serum of immunized chickens [24,25,44,45,46]. The high neutralization titer of avIBDV-SHG19 was detected by neutralization test in chickens immunized with r-*L. lactis*-avVP2-RCK. In order to verify that the neutralization titer was indeed produced by neutralizing antibodies, we prepared r-*L. lactis*-avVP2-RCK for SDS-PAGE detection; the specific protein bands of avVP2-RCK were observed in chicken serum 2 weeks after immunization as the primary antibody for Western blots, because such antibodies were not detected in ELISA kits coated with both full virus and VP2. These results suggest that r-*L. lactis*-avVP2-RCK induces a unique VP2 neutralizing antibody that is different from those produced by other vaccines. The novel neutralizing antibody provides an additional protective barrier for the prevention and control of IBDV. We speculate that this phenomenon may have multiple reasons. First, the VP2-RCK fusion protein may form a new spatial and topological structure in lactic acid bacteria, further affecting its processing and presentation pathways, and forming unique antibodies following different B cell epitopes. Alternatively, the processing and presentation of VP2 by lactic acid bacteria as a particle antigen may contribute to this phenomenon. However, further research is needed to distinguish these possibilities.

*L. lactis* is an important microorganism used in industry as a homozygous bacterium in the fermentation of many dairy products, and the host strain NZ3900 used in this study is a derivative strain of *L. lactis* MG1363 [28,47,48,49]. We expressed the VP2 protein of avIBDV in *L. lactis* strain NZ3900 and used r-*L. lactis*-avVP2-RCK for injection immunization of chickens. Since the NZ3900 strain is absent in the intestinal tract of chickens, the balance of intestinal flora would not be disturbed when the chicken immune system reacts to this strain. Additionally, the nucleic acids and cell wall components of lactic acid bacteria have good immune adjuvant activity, which promotes the immune effect of r-*L. lactis*-avVP2-RCK in chickens [28,47]. However, further studies are needed to determine whether some of the proteins in r-*L. lactis*-avVP2-RCK had any effect on chickens after injection. While screening host strains, we did not choose the lactic acid bacteria in the chicken intestine as the host strain, because we feel that immune responses of the chickens against this injected bacteria may also target intestinal probiotics, which may lead to an imbalance of intestinal flora. However, further studies are needed to prove this effect.

## 5. Conclusions

In this study, we constructed r-*L. lactis* expressing avIBDV VP2 as a subunit vaccine. We combined the RCK protein of *Salmonella enterica* and the VP2 gene of the variant IBDV within the pNZ8149 vector, transferred it into the *L. lactis* NZ3900 strain, and successfully expressed the avVP2-RCK protein. We showed that r-*L. lactis*-avVP2-RCK can induce unique and specific neutralizing antibodies against avVP2, and can completely protect chickens from avIBDV-SHG19, such that chickens challenged with vvIBDV-HLJ0504 exhibit a 100% survival rate. Additionally, after challenging with avIBDV, the cell proliferation activity of peripheral PBMCs was enhanced and the body was induced to produce high levels of IFN-γ, which reduced the body’s inflammatory response. This indicates that *L. lactis* is a potentially useful tool for vaccine development. However, the ELISA titers of serum antibodies were all negative, indicating that the immune delivery system of recombinant lactic acid bacteria is different from other conventional IBDV vaccines. It is suggested that immunization of our recombinant lactic acid bacteria can produce unique antibodies that other vaccines cannot produce, can compensate for the immune protection that other vaccines cannot produce, thus establishing a new immune protection barrier, so it is of great practical significance for IBD prevention and control. Hence, further studies are required to elaborate on the major mechanisms of immune protection of the recombinant Lactococcus.
